# Transmission of African swine fever virus to the wild boars of Northeast India

**DOI:** 10.1080/01652176.2023.2178689

**Published:** 2023-02-22

**Authors:** Lukumoni Buragohain, Nagendra Nath Barman, Suparna Sen, Arpita Bharali, Biswajit Dutta, Bhaskar Choudhury, Kuralayanapalya Puttahonnappa Suresh, Shubham Gaurav, Rakesh Kumar, Samshul Ali, Sachin Kumar, Yashpal Singh Malik

**Affiliations:** a College of Veterinary Science, Assam Agricultural University, Guwahati, Assam, India; b Wildlife Trust of India, CWRC, Kaziranga, Assam, India; c ICAR-National Institute of Veterinary Epidemiology and Disease Informatics (NIVEDI), Bengaluru, Karnataka, India; d Indian Institute of Technology, Guwahati, Assam, India; e College of Animal Biotechnology, Guru Angad Dev Veterinary and Animal Sciences University, Ludhiana, Punjab, India

**Keywords:** Pig, porcine, African swine fever, ASF, Assam, India, *B646L* gene, genotype-II, transmission, wild boar

## Abstract

**Background:**

India recorded the first outbreak of African swine fever (ASF) in North-eastern region (NER) in the year 2020.

**Aim:**

The current study was undertaken to investigate the transmission of African swine fever virus (ASFV) in the wild boars of Northeast India, particularly of Assam.

**Material and Methods:**

ASF suspected mortal tissue remains and blood samples of wild boars collected from different locations of Assam were screened for molecular detection of swine viruses which includes Classical swine fever virus, Porcine Circovirus 2, Porcine reproductive and respiratory syndrome virus and ASFV.

**Results:**

One sample each from Manas and Nameri National Parks was detected positive for ASFV. Besides this, one of the samples was positive for CSFV and one of the ASFV positive samples was also positive for PCV2. Several striking gross and microscopic alterations were noticed in different organs of ASFV infected animals. Sequencing and phylogenetic analysis of *B646L* gene confirmed the presence of ASFV genotype-II in wild boars. Circulation of similar genotype in domestic pigs of NER in the contemporary period as well as locations near to the aforementioned national parks indicates the transmission of ASFV from domestic to wild boars.

**Clinical Relevance:**

The detection of ASFV in the wild boars of Assam is alarming as it is an impending threat to pig population and other endangered species (particularly Pygmy hog), making it increasingly daunting to control the disease.

**Conclusion:**

Chances are high for ASFV to become endemic in Assam region if stringent measures are not taken at proper time.

## 1. Introduction

African swine fever (ASF) is a highly transmissible, fatal viral disease affecting wild boars and domestic pigs. ASF was first reported by Montgomery in 1921 in Kenya, following which it was detected in several regions of Africa and Eurasia (Chenais et al. 2019). This disease is caused by the African swine fever virus (ASFV), a complex, large, cytoplasmic, and icosahedral dsDNA virus classified as a unique member of the *Asfarviridae* family (Dixon et al. 2005). ASFV genome is 170-193 kbp in length with 150–167 open reading frames (Rajukumar et al. 2021). The virus presents high genetic and antigenic variability with 24 different genotypes, circulating within wild and domestic suids. The clinical signs of ASF are variable and non-specific which includes high fever, cyanosis in skin, anorexia, respiratory distress, and sudden death (Blome et al. 2020). The most common pathological changes that are encountered in ASFV infection are petechial haemorrhages in kidney, splenomegaly, enlarged liver and lymph nodes with haemorrhages (Blome et al. 2020). Microscopically, depletion of lymphocytes in lymph nodes and spleen is one of the striking characteristics of ASFV infection (Salguero et al. 2005; Nga et al. 2020; Salguero 2020).

When infected with ASFV, Bushpigs, Giant forest hogs, and Warthogs remain persistently infected without clinical signs and maintain the transmission cycle of the virus (Beltran-Alcrudo et al. 2017). Apart from transmission *via* direct contact with infected animals or fomites, the virus may also infect soft ticks of the genus *Ornithodoros* and persist in them for more than five years, acting as reservoirs (Sánchez‐Vizcaíno et al. 2012). Therefore, the epidemiology of ASF is complex and it continues to be a challenging disease. Furthermore, ASFV is highly resistant and can persist in carcasses of Wild boar and domestic pigs for long in different environmental conditions (Fischer et al. 2020). Natural materials like contaminated soil and water are also important source of ASFV. It was observed that ASFV can be infectious for about 3 months in contaminated soil (Kovalenko et al. 1965). Again, in water ASFV can be infectious for 176 days during winters whereas the virus may be active for 50 days during summer (Chenais et al. 2019). It has already been reported that contaminated river could be a putative source of ASFV transmission as several outbreaks were observed due to contaminated water from the Danube River (Boklund et al. 2018). Furthermore, the role of wild boars in the viral spread during the risk assessment analysis in commercial pig production and domestic rearing is an essential aspect that necessitates due consideration (Yoo et al. 2020).

Experimentally, it was discovered that pigs that survive the subacute infections with moderate or low virulence isolates shed the virus for at least 70 days. Hence, they may spread the virus to pigs in contact (de Carvalho Ferreira et al. 2013). Despite substantial studies toward developing a safe and efficacious vaccine to combat ASF, there are currently no commercial vaccines available against the disease (Dixon et al. 2019). Thus, continuous monitoring of the risk factors associated with transmission cycle of ASFV is vital to prevent and control the disease. The spread of ASFV from the endemic area is facilitated by legal and illegal movements of live animals and the importation of animal products, by-products, and animal feed (Kumar et al. 2020). The other important factors that are associated with transmission and outbreaks of ASFV are human knowledge and activities, social and economic factors such as tradition, cultural identity, relationship between farmers and administration, poverty level and income (Chenais et al. 2017a, 2017b; Chenais et al. 2019; Dixon et al. 2020). Human activities like mismanagement of ASFV infected carcasses by farmers in rural areas or by hunters in wild can facilitate the transmission of ASFV between wild and domestic swine populations (Thomson 1985; Jori et al. 2018; Dixon et al. 2020). Moreover, it is an established fact that Wild boar is a potent factor that is actively involved in the transmission of ASFV through Wild boar-to-pig transmission or domestic pig-to-Wild boar transmission (Guinat et al. 2016; Gaudreault et al. 2020).

India recorded the first outbreak of ASF in its North eastern (NE) region in the year 2020 (Rajukumar et al. 2021). Since the first outbreak of ASF in China in August 2018, several hundred cases of ASF have been reported in other Asian countries. The outbreak in NE India could result from the porous borders that the region shares with China, Bhutan, Bangladesh, and Myanmar (Bora et al. 2020). As the initial outbreaks of ASFV in north-eastern states of India were reported from the river banks of Brahmaputra or its tributaries or from the districts through which Brahmaputra makes its route (Bora et al. 2020; Patil et al. 2020), thus the spread of the virus through contaminated river water cannot be ruled out.

Till date, the disease has only been reported from the domestic pigs of the NE region of India, and no systematic investigation is being conducted on Wild boars of the NE region. Therefore, the current study was undertaken to investigate the transmission of ASFV in the Wild boars of Northeast India, particularly in Assam. In addition, co-infection of ASFV with other common swine viruses in Wild boars was also checked to assess the ASF situation in NE region of India accurately as there are reports of dual ASFV infection or ASFV with other viruses (Dundon et al. 2022) due to factors like high circulation of a disease, prevalence of endemic diseases, co-circulation of viral strains in a particular locality, geographically and temporally links between separate outbreaks (Mulumba-Mfumu et al. 2017; Fiori et al. 2021).

## 2. Materials and methods

### 2.1. Collection of samples

Suspected ASFV infected samples (tissue and blood) of Wild boars were received at Advanced Animal Disease Diagnosis and Management Consortium (ADMaC) laboratory, College of Veterinary Science, Assam Agricultural University, Khanapara, Guwahati, Assam, India, from different locations of Assam. The Wild boar samples were collected and provided by the Wildlife Trust of India (WTI) for diagnosis. The details of the samples collected from Wild boars are described in Table 1. Besides Wild boar samples, ASF positive tissue samples of three domestic pigs present in the repository of the ADMaC laboratory were also considered for molecular analysis to establish the probable transmission of ASFV from domestic pigs to Wild boars. Therefore, the archived domestic pig tissue samples were selected from different locations that are either close to the national parks of Assam (as mentioned in Table 1) or to the river Brahmaputra. Accordingly, two domestic pig samples were chosen from Dhemaji and Darrang districts of Assam which were collected during outbreaks in March 2020 and June 2021, respectively, and one sample was selected from Papum Pare district of Arunachal Pradesh which was collected from an outbreak that occurred in April 2020.

**Table 1.** Details of the outbreaks in wild boars.

Location	No. of affected animals	Date of outbreak onset	Virus detected (No. of animal)
Manas National Park, Assam	3	13.05.2020	ASFV and PCV2 (1)
Pobitora Wild life Sanctuary, Assam	1	11.04.2020	CSFV (1)
Nameri National Park	4	21.07.2021	None
Nameri National Park	1	18.11.2021	ASFV (1)

The tissue samples of Wild boars were divided into two parts; one part was used for molecular detection and confirmation, while the other part was preserved in 10% formalin for histopathological examinations.

### 2.2. Pathomorphological changes

Gross pathological alterations of the wild boar carcasses were recorded. For histopathological studies, respective tissue samples were collected in 10% formal saline solution, trimmed into thin pieces of about 2 mm, fixed in 10% formalin, and processed by routine alcohol-xylene dehydration and clearing method. The paraffin embedding was done by paraffin wax, and sectioning was done with a rotary microtome (ThermoScientific^TM^Shandom^TM^Finesse^TM^ 325 Manual Microtome, Runcorn, Cheshire, UK) at 4–5-micron thickness. The sections were stained with routine Haematoxylin and Eosin stain, mounted with DPX, and visualized under a light microscope (Dewinter Optical Inc, New Delhi, India) as previously described (Culling et al. 1974).

### 2.3. Detection of viral nucleic acid

The tissue samples were manually homogenized in sterile PBS (pH 7.4) using a sterile mortar and pestle to make a 10% (w/v) suspension. The tissue suspensions were pooled for each pig. Half of the suspension was used for DNA extraction, and the other half was used for RNA extraction. The total DNA was extracted from the samples using DNeasy Blood & Tissue Kits (Qiagen, Hilden, Germany), and total RNA was isolated using QIAamp Viral RNA Kit (Qiagen, Hilden, Germany). The extracted DNA was stored at −20 °C, and RNA was stored at −80 °C for further downstream processing.

Besides ASFV, the samples were also screened for other swine viruses, particularly Porcine Circovirus 2 (PCV2), Classical Swine Fever Virus (CSFV), and Porcine Reproductive and Respiratory Syndrome Virus (PRRSV). The DNA samples were used for screening ASFV and PCV2 by conventional PCR. The PCR protocol and primers used to detect ASFV were as per the OIE guidelines (Aguero et al. 2003). The PCV2 was screened by using in-house designed primers and protocol. For detection of RNA viruses, the extracted RNA was converted into cDNA by reverse transcription using the RevertAid First Strand cDNA Synthesis Kit (Thermo Fisher Scientific, Waltham, MA, USA) as per the manufacturer’s instruction. The samples were screened for CSFV by Real-Time PCR as described earlier (Rout and Saikumar 2016). Conventional RT-PCR was used to screen the samples for PRRSV as per OIE guidelines (Wernike et al. 2012). The primer details and PCR methods used are highlighted in supplementary Table TS1.

### 2.4. Phylogenetic analysis of ASFV

The ASFV positive samples were further subjected to PCR amplification of phylogenetic marker, particularly 3′ end of *B646L* gene, which encodes p72 protein using primers as described earlier (Bastos et al. 2003). The PCR products were subjected to agarose gel electrophoresis, followed by purification of the amplified DNA products using QIAquick® gel extraction kit (Qiagen, Hilden, Germany). The purified DNA products were subjected to Sanger sequencing (1st Base, Seri Kembangan, Selangor, Malaysia). The sequencing data were analysed by bioinformatics tools such as the BLASTn web server and BioEdit program.

The partial *B646L* gene sequences (3′ end) of ASFV belonging to different genotypes were retrieved from the NCBI GenBank database. The phylogenetic analysis was done using MEGA11 software (Tamura et al. 2021). Alignment of multiple sequences was carried out using the CLUSTALW program present within MEGA11. All positions that contained alignment gaps and missing data were eliminated from the analysis. The phylogenetic tree was constructed using the UPGMA method (Stefan Van Dongen and Winnepenninckx 1996) based on the Kimura-2-parameter substitution model (Kimura 1980), and the statistical significance of the phylogenetic tree was tested with 1000 bootstrap replicates.

## 3. Results

### 3.1. Gross pathology

**Figure 1.**
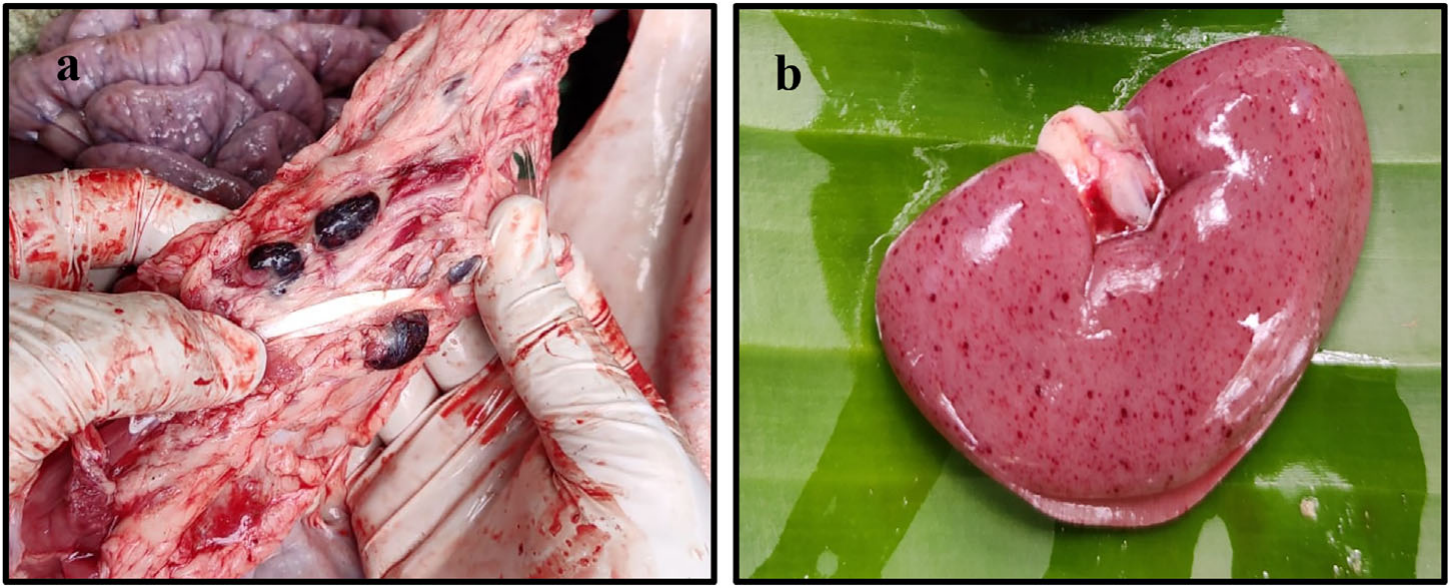
Gross alterations in ASFV infected tissues. (a) Enlarged and congested lymph nodes. (b) Extensive petechial haemorrhages in the subcapsular area of the kidney.

In gross pathological examination, out of the nine carcasses, four were in good condition and the remaining five carcasses were putrefied. In post-mortem examination, several gross alterations were observed in different organs of the affected animals. In the skin, cyanosis was observed around the ears, ventral abdomen, snout, tail, and perianal area. The gastric mucosa showed focal to diffuse areas of haemorrhages, and in some cases, there was focal accumulation of blood. Severe haemorrhages were observed in the mucosa of the small intestine. There was accumulation of straw or blood-tinged fluid in the abdominal cavity. The hepatic parenchyma showed focal to diffuse areas of necrosis with distention of the gall bladder with bile. Splenomegaly was quite prominent. Enlargement and congestion of varying degrees were recorded in the lymph nodes (Figure 1a). The renal parenchyma showed petechial haemorrhages on the surface (Figure 1b). Lung parenchyma showed varying degrees of consolidation and oedema. In the brain, congestion of cerebral blood vessels was recorded. The gross alterations observed during autopsy in this study indicated possible acute or subacute cases of ASF.

### 3.2. Histopathological alterations

**Figure 2.**
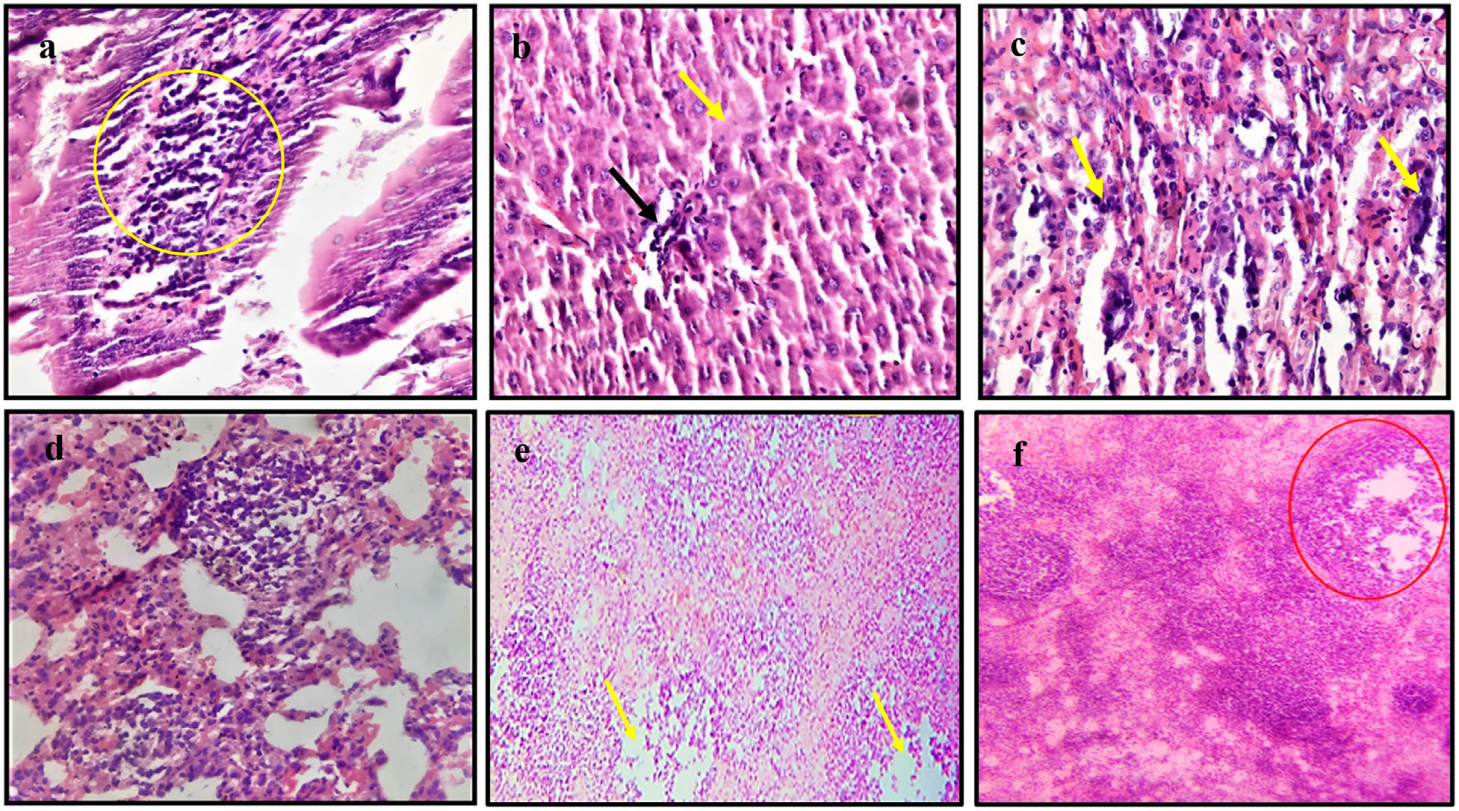
Histopathological examination of ASFV infected tissues. (a) Intestine showing infiltration of lymphocytes in the submucosa (magnification 40 times, (b) Liver showing necrosis (yellow arrow) with lymphoplasmacytic infiltration (black arrow)(40×), (c) Kidney showing aggregation of mononuclear cells (yellow arrow) with haemorrhages (40×), (d) Lung showing accumulation of inter and intra alveolar aggregation of mononuclear cells, predominantly macrophages (40×), (e) Lymph node showing depletion of lymphocytes from lymphoid follicle (yellow arrow) with accumulation of necrotic debris (10×), and (f) Spleen showing depletion of lymphocytes from the splenic corpuscles (red circle)(10x).

During the histopathological investigation, several characteristic microscopic alterations were observed in various tissues of ASFV infected wild boars (Figure 2a–f). The intestinal mucosa showed catarrhal enteritis characterized by hyperaemia, goblet cell hyperplasia, and infiltration of lymphocytes and neutrophils. The hepatic parenchyma showed centrilobular degenerative changes with focal areas of necrosis. Small foci of lymphoplasmacytic infiltrate in hepatic sinusoids with enlarged Kupffer cells were also seen. The splenic parenchyma showed depletion of lymphocytes from the splenic white pulp. The splenic red pulp appeared full of mononuclear cells, cell debris, and fibrin deposits. The lymph nodes showed congestion of the capillaries with focal areas of haemorrhages. The depletion of lymphocytes from the lymphoid follicles was prominent. The renal parenchyma showed degeneration of the tubular epithelium with focal to diffuse areas of coagulative necrosis. In some instances, focal aggregation of the mononuclear cells, predominantly lymphocytes, and macrophages, was recorded with or without cystic dilatation. Interstitial haemorrhages in the renal cortex, medulla and renal pelvis were also seen. The lung parenchyma showed thickening of interalveolar septa due to the infiltration of macrophages. Apart from this, scattered mild alveolar haemorrhages, diffuse moderate alveolar oedema along with the presence of cell debris, fibrin deposits, and macrophage infiltration were also observed. The cardiac muscles showed various stages of degeneration like karyorrhexis, pyknosis, and chromatolysis as well as a varying degree of congestion and haemorrhages were also observed.

### 3.3. Molecular detection and phylogenetic analysis

In molecular screening, it was observed that two of the wild boar samples received from Manas National Park and Nameri National Park were positive for ASFV. The sample received from Pobitora Wildlife Sanctuary was found to be positive for CSFV. Additionally, the sample received from Manas National Park was also found positive for PCV2. However, none of the wild samples were found to be positive for PRRSV.

**Figure 3.**
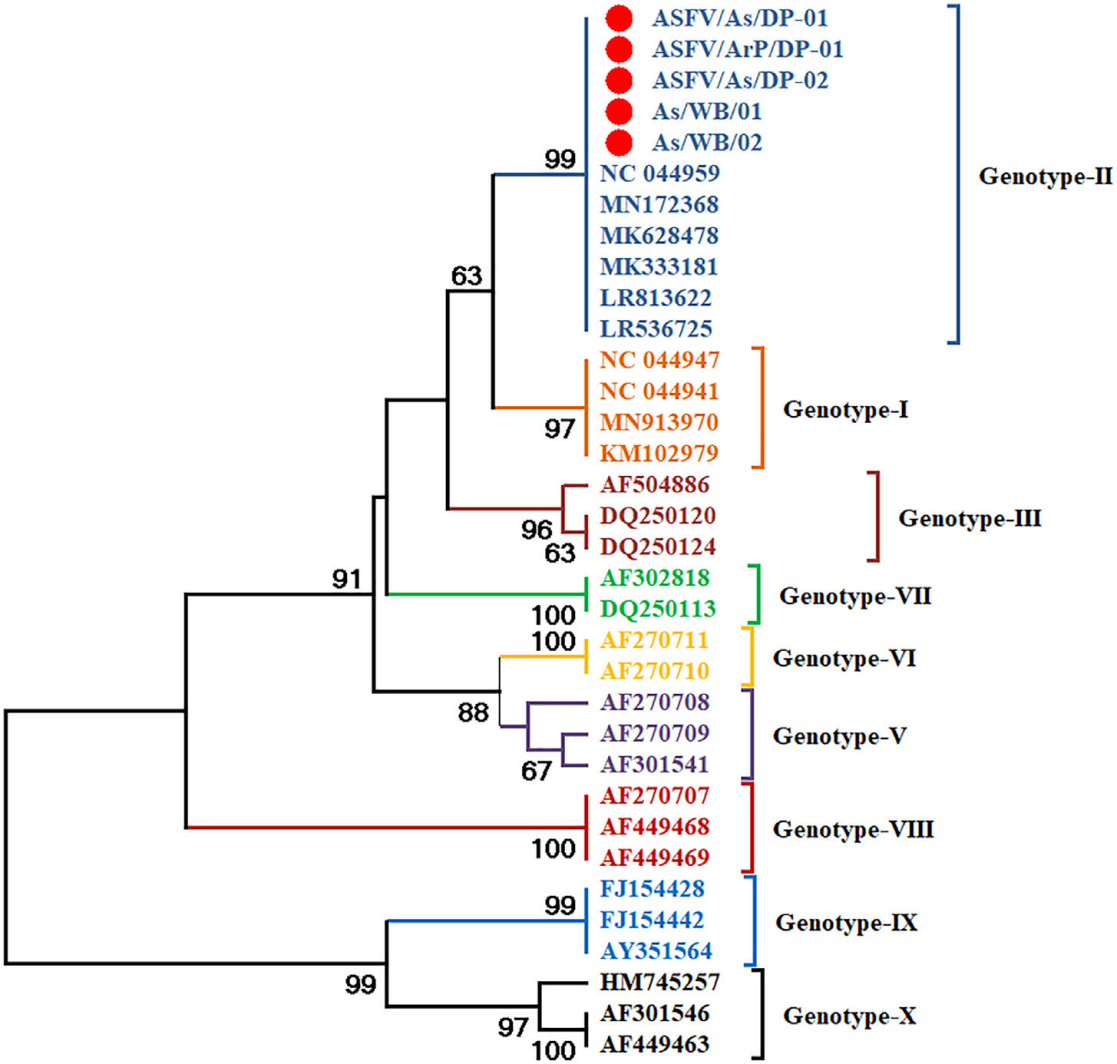
Phylogenetic tree of ASFV based on partial *B646L* gene. The ASFV strains detected in wild boars and domestic pigs (highlighted with red circle) formed clade with genotype-II strains (dark blue colour clade) of ASFV.

The partial *B646L* gene (478 bp) of two ASFV positive samples was successfully amplified and sequenced. Similarly, 478 bp of the *B646L* gene from three domestic pig samples were amplified and sequenced. The sequencing data were further analysed using the web server BLAST and offline tool BioEdit. Phylogenetic analysis done in MEGA 11 software suggested that the ASFV strains circulating in Manas National Park (As/WB/01) and Nameri National Park (As/WB/02) of Assam belongs to the genotype-II (Figure 3). Moreover, the ASFV detected in three domestic pig samples (strain from Arunachal Pradesh: ASFV/ArP/DP-01 and strains from Assam: ASFV/As/DP-01 and ASFV/As/DP-02) were also of the same genotype II which supports the transmission of ASFV from domestic pigs to wild boars.

## 4. Discussion

African swine fever virus infection can produce a variety of clinical presentations ranging from chronic, subclinical, or low-level disease to haemorrhagic fever and peracute death. In ASFV infection, similar kind of clinical signs and pathological alterations are observed in wild boars and domestic pigs. In this study, the detection of ASFV in wild boars of North-eastern region of India has been reported and the associated genotype has been revealed.

In the gross pathological examination, the lesions recorded in domestic pigs and wild boars due to ASFV infection are of alike nature (Pérez et al. 1998; Gabriel et al. 2011; Salguero 2020) and the gross lesions observed in this study are similar to those described in European wild boar which were reviewed previously (Blome et al. 2013; Pikalo et al. 2019; Sauter-Louis et al. 2021a). Similar type of cyanosis in different parts of body skin were reported in ASFV infected pigs (Sánchez-Vizcaíno et al. 2015; Salguero 2020). Petechial haemorrhages are not uncommon in intestines of ASFV infected pigs (Sánchez-Vizcaíno et al. 2015; Nga et al. 2020; Salguero 2020). Extensive studies on pathological changes in ASFV infected kidney also observed similar types of haemorrhages on the surface of kidney (Gómez-Villamandos et al. 1995; Hervás et al. 1996; Sánchez-Vizcaíno et al. 2015). Lung parenchyma showed varying degrees of consolidation and oedema and alike lesions were described by Salguero (2020) in ASFV infection. The gross alterations observed during autopsy in this study indicated possible acute or subacute cases of ASF (Nga et al. 2020; Salguero 2020) as there was prominent splenomegaly (Konno et al. 1972), cyanosis in different parts of the skin (Salguero et al. 2002), varying degree of haemorrhages in lymph nodes, kidneys and gastrointestinal tract (Hervás et al. 1996; Carrasco et al. 1997; Sánchez-Vizcaíno et al. 2015; Nga et al. 2020; Salguero 2020).

Immunosuppression due to lymphoid depletion (caused by apoptosis of lymphocytes) is one of the striking characteristics of ASFV infection (Oura et al. 1998; Salguero et al. 2005; Nga et al. 2020; Salguero 2020). Depletion of lymphocytes was observed in lymphoid organs like the spleen and lymph node which is similar to earlier observations (Salguero et al. 2002; Salguero et al. 2005). Like previous studies, the splenic red pulp was filled with mononuclear cells, cell debris, and fibrin deposits (Carrasco et al. 1997; Salguero 2020). The microscopic changes that were recorded in the lung, liver, heart, and kidney of wild boars were similar to that observed in previous ASFV infections in pigs (Konno et al. 1971; Carrasco et al. 1997; Yabe et al. 2015; Semerjyan et al. 2018; Nga et al. 2020; Salguero 2020).

This is the first report of molecular detection and confirmation of ASFV in wild boars from north-eastern states of India. However, ASFV have been detected in wild boars from several countries of Asia and Europe (Li et al. 2019; Pikalo et al. 2019; Kim et al. 2020; Sauter-Louis et al. 2021a; Sauter-Louis et al. 2021b). In an experimental infection in European wild boars the major visceral organs and lymph nodes exhibiting pathological lesions were found to be positive for ASFV genome in Real-Time PCR (Gabriel et al. 2011). Similar type of macroscopic and microscopic pathological alterations in different tissue samples was recorded in ASFV confirmed cases by PCR and Real-Time PCR assays (Nga et al. 2020). In Tanzania, the tissue samples of pigs positive for ASVF in PCR also exhibited haemorrhages in heart, kidney, spleen and several lymph nodes during post-mortem examination (Yona et al. 2020). Organs like spleen, kidney, liver, lymph nodes and colon exhibiting several pathological alterations in autopsy were found to be ASFV positive in Real-Time PCR (Lee et al. 2021). Recently, from Mizoram state of India also described haemorrhagic lesions in visceral organs of domestic pigs which during post-mortem examination were found to be ASFV positive in molecular analysis (Rajkhowa et al. 2022). Earlier, Blome et al. (2012) reported the possible transmission of ASFV from domestic pigs to European wild boars. The report of India’s first ASF outbreak in 2020 has confirmed that the ASFV causing outbreaks in Assam was genotype-II (Rajukumar et al. 2021).

**Figure 4.**
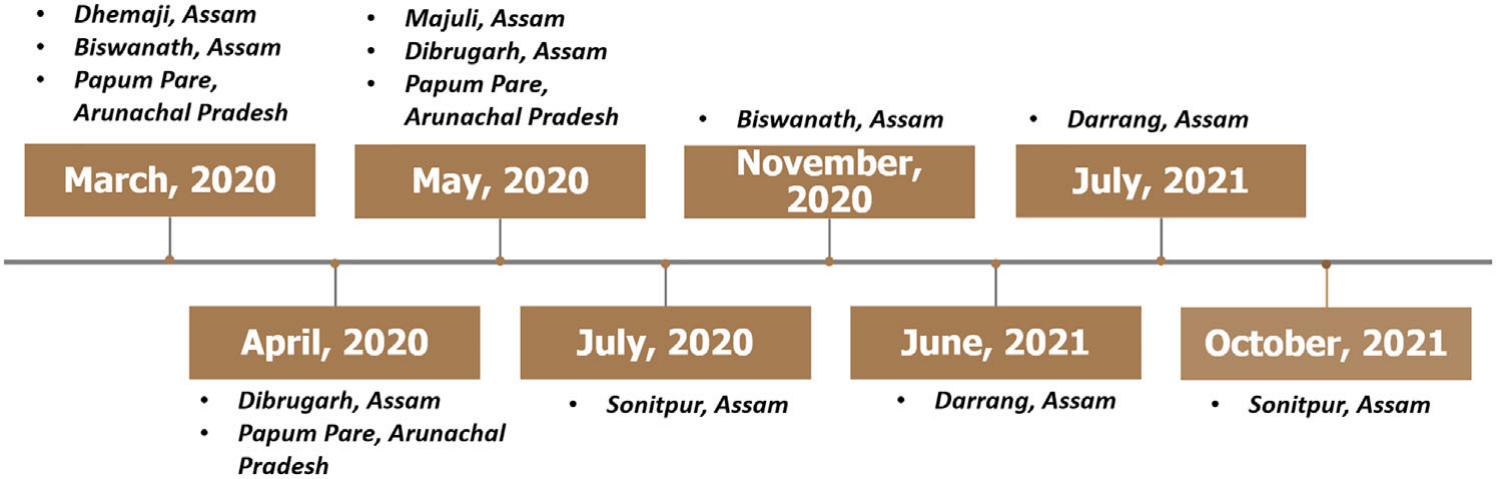
Timeline of ASFV outbreaks. Timeline of suspected ASFV outbreaks in Assam and Arunachal Pradesh which might be responsible for the probable transmission of ASFV from domestic pigs to wild boars of Assam.

**Figure 5.**
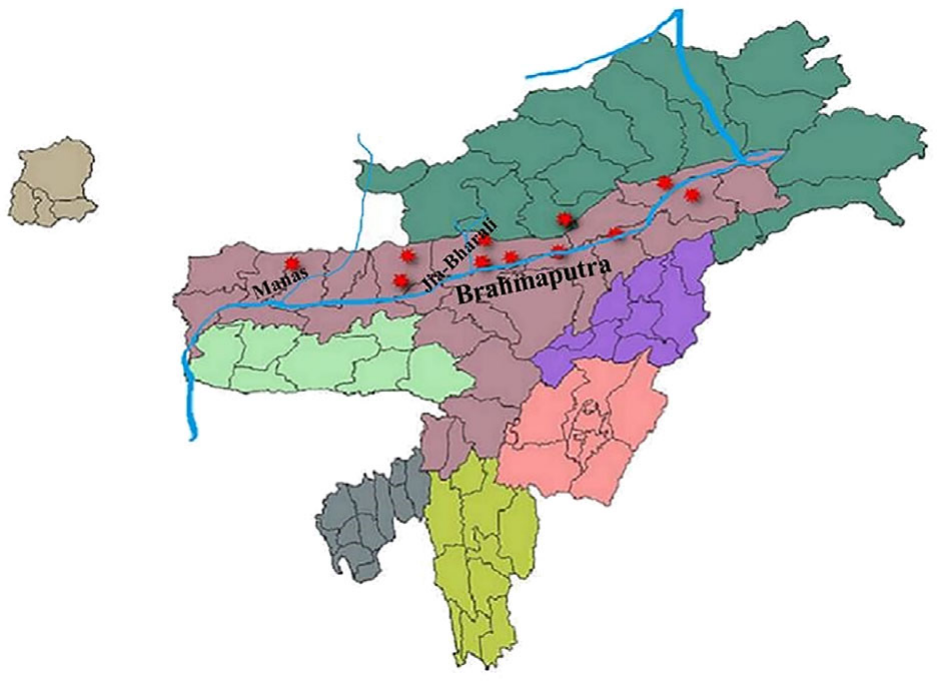
Map of North-eastern states of India representing ASFV outbreaks in the national parks (Manas and Nameri) and the places near the river Brahmaputra where outbreaks were reported. The tributaries (Manas and Jia-Bharali rivers) of Brahmaputra that passes through the two national parks are also depicted in the map. Outbreaks at different locations are highlighted with red marker.

There are several ASFV outbreaks since March, 2020 that occurred in domestic pigs of Assam and Arunachal Pradesh (Figure 4) which might be a potent source of transmission of ASFV from domestic pigs to wild boars. The probable transmission of ASFV to the wild boar of the Manas National Park might be through Manas River, which is one of the major tributaries of the mighty Brahmaputra passing through the heart of the national park. Since most of the initial outbreaks in Assam were reported from the banks of Brahmaputra or alongside its tributaries which might be due to the disposal of infected carcass in rivers (Bora et al. 2020). Again, the transmission of ASFV into the Nameri National Park might be due to the outbreaks that occurred at nearby locations of the national park which were at Darrang district in June and July 2021, and Sonitpur in September, 2021 (Figure 4). However, the source of infection through the porous border between Arunachal Pradesh and the National Park cannot be ignored because there were ASF outbreaks in the Arunachal Pradesh state since 2020 (Bora et al. 2020; Rajukumar et al. 2021). Therefore, one of the domestic pig samples was also chosen from the state of Arunachal Pradesh. Furthermore, one of the major rivers (Jia-Bharali) flow through this national park, and there are a few tributaries of this river that originated in Arunachal Pradesh (Figure 5). Thus, entry of the ASFV to Nameri National Park through contaminated river cannot be ruled out. Contaminated water is a potent source of ASFV infection (Chenais et al. 2019) and contaminated river water has link with ASFV outbreaks in nearby areas through which the river flows (Boklund et al. 2018).

This study is the first report of ASFV infection in wild boars of India belonging to genotype-II. However, ASFV was detected in wild boar of China in 2018, which was confirmed to be genotype-II based on partial B646L gene sequence and serogroup 8 based on CD2v (Li et al. 2019). In 2019, South Korea reported the first case of ASF in wild boar that also belongs to genotype-II based on p72 encoding gene (Kim et al. 2020). ASFV genotype-II was detect and confirmed in wild boars of Germany in 2020 which was suspected to entered from Poland (Sauter-Louis et al. 2021b). The reason for the introduction of ASFV to the North-eastern states of India is suspected to be the porous international border (Bora et al. 2020) along with other risk factors such as smuggling of pigs, illegal import and export of pork and pork products, etc (Kumar et al. 2020). Most of the outbreaks that occurred in Asia, particularly in China, were found to be caused by genotype-II of ASFV (Ge et al. 2018; Kim et al. 2020; Mai et al. 2021), which further bolsters the suspicion of ASFV entry into NE India from neighbouring countries. However, the recent report on genotype-I ASFV from China (Sun et al. 2021) is another emerging threat to the piggery industries in Asian countries.

PCV2 is another DNA virus that infects both wild and domestic pigs and causes immunosuppression (Opriessnig and Halbur 2012; Meng 2013; Beltran-Alcrudo et al. 2017; Ramos et al. 2017). Co-infection of PCV2 with other swine viruses is one of the key factors that lead to Porcine circovirus-associated disease (Opriessnig and Halbur 2012; Beltran-Alcrudo et al. 2017; Ouyang et al. 2019). Furthermore, PCV2 can also enhance the infection of other swine viruses by increasing their rate of replication and subsequent infection (Ouyang et al. 2019). The possibility of ASFV and PCV2 co-infection has already been reported from the archived DNA samples in Indonesia and Mongolia (Dundon et al. 2022). In the wild boar, PCV2 might persist without exhibiting prominent clinical or pathological changes and can be a potent reservoir (Ramos et al. 2017). However, ASFV super-infection could be a triggering factor for disease progression in the wild boar and may be aggravating the ASFV infection due to rapid replication of ASFV because PCV2 infection increases the replication of other pathogens (Ouyang et al. 2019).

In conclusion, the presence of ASFV with PCV2 in the wild boars of Assam is an alarming factor not only for the domestic pigs but also for one of the engendered species, i.e. the Pygmy hog, which is found mostly in Assam with a very limited population. A probable explanation for the detection of ASFV in the wild could be the transmission of the virus from the affected domestic pigs of the adjoining regions through different routes, albeit other possible modes of transmission cannot be ruled out which needs further investigation. The entrance of ASFV into the wild is likely to make it more complicate to control the disease. Furthermore, there are high chances of ASFV being endemic in this region of India if strict measures are not undertaken immediately.

## Supplementary Material

Supplemental Material
