# Supplementary material for: Transmission of African swine fever virus to the wild boars of Northeast India
Source: Vet Q. 2023 Feb 22;43(1):1–10. doi: 10.1080/01652176.2023.2178689 (PMC10124978; doi:10.1080/01652176.2023.2178689)
Supplement: Supplemental Material [file TVEQ_A_2178689_SM8926.docx]

***Supplementary data***

**Transmission of African Swine Fever Virus to the Wild Boars of Northeast India**

Lukumoni Buragohain^1^, Nagendra Nath Barman^1*^, Suparna Sen^1^, Arpita Bharali^1^, Biswajit Dutta^1^, Bhaskar Choudhury^2^, Kuralayanapalya Puttahonnappa Suresh^3^, Shubham Gaurav^4^, Rakesh Kumar^4^, Samsul Ali^2^, Sachin Kumar^4^, Yashpal Singh Malik^5^

^1^College of Veterinary Science, Assam Agricultural University, Guwahati, Assam, India

^2^Wildlife Trust of India, CWRC, Kaziranga, Assam, India

^3^ICAR-National Institute of Veterinary Epidemiology and Disease Informatics (NIVEDI), Bengaluru, Karnataka, India

^4^Indian Institute of Technology, Guwahati, Assam, India

^5^College of Animal Biotechnology, Guru Angad Dev Veterinary and Animal Sciences University, Ludhiana, Punjab, India

***Corresponding author:** Nagendra Nath Barman. College of Veterinary Science, Assam Agricultural University, Guwahati, Assam, India. **Email:** [nnbarman@gmail.com](mailto:nnbarman@gmail.com).

**Supplementary Table TS1:** Primers and PCR methods for detection of ASFV, PCV2, CSFV and PRRSV and sequencing of *B646L* (p72) gene of ASFV for genotyping

Aguero, M., Fernández, J., Romero, L., Sánchez Mascaraque, C., Arias, M., & Sánchez-Vizcaíno, J. (2003). Highly sensitive PCR assay for routine diagnosis of African swine fever virus in clinical samples. *Journal of Clinical Microbiology, 41*(9), 4431-4434.

Bastos, A. D., Penrith, M.-L., Cruciere, C., Edrich, J., Hutchings, G., Roger, F., . . . R Thomson, G. (2003). Genotyping field strains of African swine fever virus by partial p72 gene characterisation. *Archives of Virology, 148*(4), 693-706.

Rout, M., & Saikumar, G. (2016). Development and application of real-time TaqMan RT-PCR assay for improved detection of classical swine fever virus in slaughtered pigs. *Indian Journal of Animal Research, 50*(6), 979-982.

Wernike, K., Bonilauri, P., Dauber, M., Errington, J., LeBlanc, N., Revilla-Fernández, S., . . . Beer, M. (2012). Porcine reproductive and respiratory syndrome virus: interlaboratory ring trial to evaluate real-time reverse transcription polymerase chain reaction detection methods. *Journal of Veterinary Diagnostic Investigation, 24*(5), 855-866.

**References:**

| **Virus** | **Primer Name** | **Sequence (5’🡪3’)** | **Method** | **References** |
| --- | --- | --- | --- | --- |
| CSFV | CSFL1 | TGGGTGGTCTAAGTCCTGAGT | Real Time RT-PCR, (SYBR Green Chemistry) | Rout and Saikumar, 2016 |
|  | CSFR1 | GTGTGATTTCACCCTAGCGA |  |  |
| PCV2 | PCV-RepF | AAGTGAGCGGGAAAATGC | Conventional PCR | In-house designed |
|  | PCV-RepR | GGAAATTCAGGGCATGGG |  |  |
| PRRSV | Forward | ATGGCCAGCCAGTCAATCA | Conventional RT-PCR | Wernike et al., 2012 |
|  | Reverse | TCGCCCTAATTGAATAGGTGACT |  |  |
| ASFV | PPA-1 | AGTTATGGGAAACCCGACCC | Conventional PCR | Agüero et al., 2003 |
|  | PPA-2 | CCCTGAATCGGAGCATCCT |  |  |
| ASFV (Genotyping primers) | P72-U | GGCACAAGTTCGGACATGT | Conventional PCR | Bastos et al., 2003 |
|  | P72-D | GTACTGTAACGCAGCACAG |  |  |
